# Patterns of Bisphosphonates Utilization in Patients under Age 45 in a Large Cohort of Commercial Insurance Beneficiaries in the United States

**DOI:** 10.1371/journal.pone.0115091

**Published:** 2015-01-22

**Authors:** Jing Xie, Angela Tong, Seoyoung C. Kim

**Affiliations:** 1 Division of Pharmacoepidemiology and Pharmacoeconomics, Department of Medicine, Brigham and Women’s Hospital, Harvard Medical School, Boston, Massachusetts, United States of America; 2 Division of Rheumatology, Immunology and Allergy, Department of Medicine, Brigham and Women’s Hospital, Harvard Medical School, Boston, Massachusetts, United States of America; Tel Aviv Sourasky Medical Center, ISRAEL

## Abstract

**Background:**

The effectiveness and safety of bisphosphonates treatment used in the young population have not been well studied. Despite insufficient data on effectiveness and safety of bisphosphonates in young patients, bisphosphonates are still considered in younger patients at high risk for osteoporosis or fracture. The objectives of this study were to identify bisphosphonate initiators aged 10–45 years and describe their clinical characteristics and to assess time trends of bisphosphonate use over the past decade in a large U.S. population-based cohort.

**Methods:**

Using the medical and pharmacy claims data from a U.S. commercial insurance (2003–2012), patients aged 10–45 years without malignancy who initiated an oral or intravenous bisphosphonate after at least 1 year of insurance enrollment were selected. Baseline demographics, comorbidities, medications and health care utilization were assessed in the year prior to initiating a bisphosphonate. The trend of bisphosphonate use over time was examined.

**Results:**

There were 9,082 bisphosphonate initiators (0.02% of the same age group in the population). The mean age was 38.1 years and 79.6% female. Osteoporosis was the most common diagnosis (41.2%). At baseline, 10.8% had a diagnosis of fracture and 29.0% had a bone mineral density measured. Of those who used glucocorticoids (39%) at baseline, the mean 1-year cumulative prednisone-equivalent dose was 2,669 milligrams. The use of bisphosphonates in the young population significantly decreased over the past decade (p<0.001).

**Conclusions:**

Among young patients aged 10–45, the use of bisphosphonates was uncommon and significantly decreased over the past decade in the U.S. While most patients initiating bisphosphonates had a diagnosis of osteoporosis and fracture in the preceding year, some had no recorded claims with a diagnosis of fracture, osteoporosis, or long-term glucocorticoids use at baseline. Future research is needed to examine the effectiveness and safety of bisphosphonates in young patients at risk for osteoporosis.

## Introduction

More than one million patients had at least one claim for oral bisphosphonate prescription in the US between 2001 and 2007.[1] Bisphosphonates are widely used as the first line therapy for osteoporosis in postmenopausal women and older men based on efficacy data in reducing the risk of osteoporotic fracture from randomized clinical trials.[2–4] However, data from randomized controlled studies are not available for adolescents and young adults due to feasibility and potential ethical issues.

Over the past decade, increasing data suggest potential safety issues associated with bisphosphonates such as osteonecrosis of the jaw, atrial fibrillation and atypical femur fracture.[5] The positive effects of the bisphosphonates treatment may be counterbalanced by a decrease in tissue heterogeneity, which could impair tissue mechanical properties.[6] To date, long-term effects of bisphosphonates are not fully understood, [7] and the effects of bisphosphonates may be different in developing bones in adolescents [8, 9]. Furthermore, there are safety concerns for women of child bearing age, because animal studies suggest bisphosphonates as small molecules may pass through the placenta and adversely affect fetal skeleton.[10] Use of bisphosphonates in women of child bearing age may be particularly problematic as a large portion of the absorbed bisphosphonates dose is bound to bone with an estimated elimination half-life of greater than 10 years, [11] and thus pre-pregnancy administration of bisphosphonates could potentially result in embryonic exposure.

Long-term use of glucocorticoids is associated with morbid complications including a decline in bone mineral density (BMD), subsequent osteoporosis, and resultant fractures. The literature suggests an increased risk of fractures is reported with a daily dosage of prednisolone or equivalent as low as 2.5–7.5 mg. [12] However, glucocorticoids use alone may not be a sufficient reason for prescribing bisphosphonates in younger patients. The American College of Rheumatology (ACR) 2012 recommendations for the prevention and treatment of glucocorticoid-induced osteoporosis suggest women with childbearing potential should have prevalent fragility fracture in addition to more than 3 months glucocorticoids use as an indication for bisphosphonates treatment. [12]

There is not yet clear treatment guideline for osteoporosis in premenopausal women and younger men due to limited data on the absolute risk of osteoporotic fracture and effectiveness and safety of bisphosphonates in these patients. It is also unknown how commonly bisphosphonates are prescribed, which bisphosphonates are preferred, and what medical conditions or medications that bisphosphonate users commonly have in adolescents and young adults. We therefore undertook a large population-based cohort study to identify incident bisphosphonate users aged 10–45 years and describe their characteristics and to assess time trends of bisphosphonate use over the past decade.

## Materials and Methods

### Data Source

We conducted a cohort study using the claims data for the period January 1, 2003 to December 31, 2012, from United HealthCare, a commercial U.S. health plan, which insures primarily working adults and their family members. This database contains longitudinal claims information including medical diagnoses, procedures, hospitalizations, physician visits, and pharmacy dispensing on its approximately 14 million subscribers across all states in the U.S. on a yearly basis. Demographics of people in the database are similar to the U.S. general population for all ages less than 65 years, whereas the geographic distribution reflects the region-specific market share of the health plan rather than the underlying population density. Patient informed consent was not required as the dataset was de-identified to protect subject confidentiality. The study protocol was approved by the Institutional Review Board of the Brigham and Women’s Hospital.

### Study Cohort

The cohort is defined as all bisphosphonate initiators between 10 and 45 years of age on the date of their first fill defined as the index date with a minimum of 12 months of enrollment prior to the index date. An initiator was defined as having not used any generics or brand names of bisphosphonate for the past 12 months. Bisphosphonates, either oral or intravenous, include risedronate, ibandronate, zoledronate, etidronate, and pamidronate. Patients with any malignancies prior to the index date were excluded.

### Data Analysis

Baseline characteristics and health care utilization factors of the cohort were assessed 12 months preceding and including the index date. Age and sex were extracted from the individual’s enrollment file and comorbidities were defined by appropriate ICD-9 CM codes and/or medication dispensing records. Cumulative prednisone-equivalent doses were calculated for systemic glucocorticoids over 3 and 12 months prior to initiation of bisphosphonates. Descriptive statistics including proportions for categorical variables and means and standard deviations for continuous variables were used to characterize the study cohort. A linear regression assessed the trends of bisphosphonate use over the study period. A two-sided p-value less than 0.05 was considered statistically significant. All analyses were done using SAS 9.2 Statistical Software (SAS Institute Inc., Cary, NC).

## Results

### Cohort Selection

During the study period, 12,568 bisphosphonates initiators were initially identified. 3,486 patients were excluded due to previous malignancies. In total, 9,082 patients were included in analysis.

### Patient Characteristics

The Table 1 shows the baseline characteristics of 9,082 bisphosphonate initiators aged 10–45 years. The most commonly used bisphosphonate was alendronate (53.5%), followed by risedronate (29.8%), ibandronate (14.3%), zoledronic acid (1.2%), and etidronate or pamidronate (1.2%). Most users were female (79.6%) and had frequent visits to physicians (15.2±14.6) [mean±SD] and high number of prescription drugs (9.4±6.9) in the 12 months prior to the index date. Osteoporosis (41.2%) was the most common comorbidity, followed by hyperlipidemia (25.2%), anxiety (17.8%), hypertension (17.7%), and depression (17.4%). In the year prior to starting a bisphosphonate, 10.8% had a diagnosis of fracture and 29.0% had a BMD measured. 14% patients used hormone replacement therapy (HRT) and 11.9% were on oral contraceptives prior to initiating a bisphosphonate.

**Table 1:** Characteristics of 9,082 patients in the year prior to initiating a bisphosphonate.

	**Mean ± SD or N (percentage)**
**Bisphosphonate Types**	
Alendronate	4,861 (53.5%)
Risedronate	2,704 (29.8%)
Ibandronate	1,303 (14.3%)
Zoledronate	110 (1.2%)
Etidronate or pamidronate	104 (1.2%)
**Demographic**	
Age (years)	38.1 ± 6.9
10–24	574 (6.3%)
25–34	1,650 (18.2%)
35–45	6,858 (75.5%)
Female	7,230 (79.6%)
**Bisphosphonate indications**	
Osteoporosis	3,743 (41.2%)
Any fracture	981 (10.8%)
Inflammatory arthritis	651 (7.2%)
Systemic lupus erythematosus	539 (5.9%)
Inflammatory bowel disease	503 (5.5%)
Other bone diseases ^a^	96 (1.0%)
**Comorbidities**	
Hyperlipidemia	2,287 (25.2%)
Anxiety or sleep disorder	1,615 (17.8%)
Hypertension	1,604 (17.7%)
Depression	1,583 (17.4%)
Thyroid disease	1,403 (15.4%)
Myopathy	910 (10.0%)
Diabetes	639 (7.0%)
**Medications**	
Systemic glucocorticoids	3,538 (39.0%)
Antidepressants	2,060 (22.7%)
Proton pump inhibitors	1,603 (17.7%)
Other hormonal agents/HRT	1,276 (14.0%)
Sedatives	1,241 (13.7%)
Oral contraceptives	1,085 (11.9%)
Immunosuppressive drugs	834 (9.2%)
Anti-epileptics	712 (7.8%)
Inhaled steroids	404 (4.4%)
Antipsychotics	241 (2.7%)
**Health Care Utilization**	
No. of outpatient visits	15.2 ± 14.6
No. of specialty visits	10.9 ± 12.5
No. of prescription drugs	9.4 ± 6.9
Emergency room visit	1,270 (14.0%)
Acute hospitalization	1,764 (19.4%)
Bone density scan	2,632 (29.0%)

^a^ Includes Paget’s disease of bone, osteogenesis imperfecta, and osteomalacia.

HRT = hormone replacement therapy

Thirty-nine percent used systemic glucocorticoids at baseline with an average 12-month cumulative prednisone-equivalent dose [mean±SD] of 2,669±11,545 milligrams. The 3-month cumulative prednisone-equivalent dose [mean±SD] was 1,486±4,995 milligrams, which is approximately 17 milligrams per day of prednisone for 90 days. Use of prescription drugs such as antidepressants (22.7%), proton pump inhibitors (17.7%), and other hormones (14%) were common.

### Trend in Bisphosphonate Use

**Figure 1:**
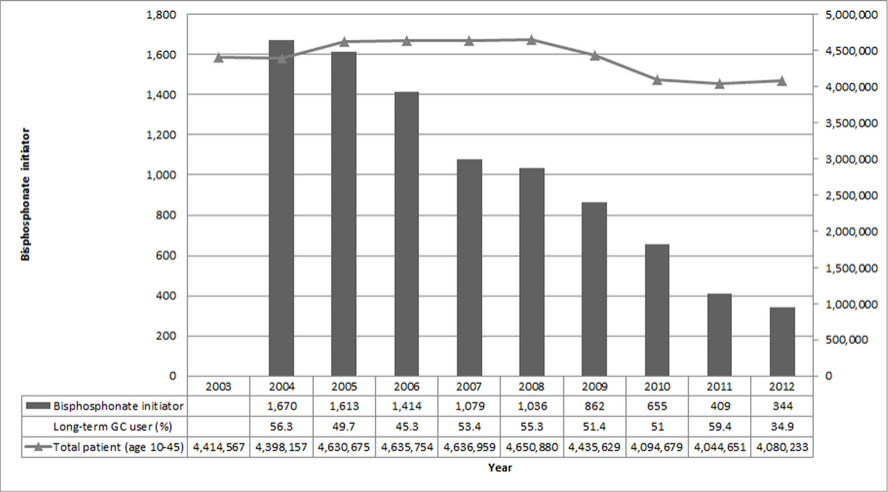
The annual relative contribution to the 9,082 bisphosphonate initiators identified over the entire period of interest (2003–2012). The dotted line represents the total number of patients aged 10 to 45 years in the study database.

The number of bisphosphonate initiators significantly declined each year from 1,670 in 2004 to 344 in 2012 (p<0.001), despite the similar number of all patients aged 10–45 in the study database (Fig. 1). Among all bisphosphonates initiators, the percentage of alendronate use fluctuated between 46% and 63%; use of etidronate and pamidronate remained less than 1% over time; whereas risedronate use decreased dramatically from 43.4% in 2004 to 15.7% in 2012. Since ibandronate’s introduction in 2005, its use increased fast and topped at 27% in 2007, and then decreased slowly to 14.8% in 2012. Since zoledronate’s introduction in 2008, its use gradually increased to 5.8% in 2012. Among all bisphosphonate initiators in 2004 (Table 2), 941 (56%) patients had long-term use of glucocorticoid in the year prior to the index date. In total, 79% bisphosphonate initiators had at least one potential bisphosphonate indication, including osteoporosis (40%), any fracture (11%), inflammatory arthritis (9%), systemic lupus erythematosus (9%), inflammatory bowel disease (9%), and other bone diseases (1%). The percentage of these diagnoses among bisphosphonate initiators remained stable over the study period.

**Table 2:** Potential indications for initiating bisphosphonate treatment.

			**Potential indications for initiating bisphosphonate treatment (%)**						
	**Number of bisphosphonate initiators**	**Number of GC users in the study database**	**Osteoporosis**	**Any Fracture**	**Arthritis**	**SLE**	**Bowel Disease**	**Other Bone Disease**	**GC Users**
2004	1,670	379,492	40.2	11.5	8.7	8.7	9.0	1.0	56.3
2005	1,613	385,374	41.9	11.7	8.4	7.2	7.9	1.2	49.7
2006	1,414	397,942	42.2	10.4	8.8	7.4	8.3	1.2	45.3
2007	1,079	392,526	47.2	12.0	11.3	8.8	7.6	1.1	53.4
2008	1,036	410,359	49.3	12.5	10.7	9.7	8.1	1.6	55.3
2009	862	430,834	44.9	13.2	10.9	10.0	7.2	1.5	51.4
2010	655	410,074	46.1	12.2	9.3	8.4	6.0	1.2	51.0
2011	409	403,453	51.6	17.1	14.9	11.5	11.2	2.0	59.4
2012	344	391,152	39.8	12.5	8.1	7.3	6.7	0.9	34.9

## Discussion

This large population-based cohort study showed that the use of bisphosphonates was uncommon in adolescents and young adults in the U.S. and significantly decreased over time. Furthermore, this study suggests a potential gap in understanding clinical practice patterns in treating young patients at a possible risk for osteoporosis, as some bisphosphonate initiators were with no clear indication for bisphosphonate treatment recorded in the year prior to initiating a bisphosphonate.

Unlike well-established diagnosis and treatment of osteoporosis in postmenopausal women and older men, there is uncertainty in the management of osteoporosis among young patients. To date, little is known whether benefits of bisphosphonates in preventing bone loss would outweigh other potential risks among the young patient population. We also lack the evidence regarding when treatment should stop. Some experts suggest a drug holiday should be considered after several years of treatment, although there is no official recommendation to guide clinicians.[13] Use of bisphosphonates should be avoided for certain patient groups, such as pregnant women or women during lactation due to the lack of safety data in pregnancy.

From Fig. 1, we observed the number of bisphosphonates initiators per year among young patients (aged 10 to 45) decreased significantly from 1,670 to 344 over the follow-up period. This trend does not appear to be related to changes in the number of total patients or long-term GC users in the study database, since it remained relatively stable compared to the number of bisphosphonates initiators per year. Although it is speculative, this downward trend in use of bisphosphonates may be related to growing concerns over side effects of bisphosphonates over the past several years.

Systemic glucocorticoids use is common in any age group, [14] and glucocorticoid-induced osteoporosis (GIOP) is a major side effect among long-term glucocorticoids users. As the ACR 2010 Recommendations for the Prevention and Treatment of GIOP focuses mainly on older patients, [12] it is unclear how to best prevent bone loss and future fracture among younger patients at risk for osteoporosis. Current guidelines recommend a pharmacologic treatment including bisphosphonates only for patients exposed to long-term glucocorticoids who have a fragility fracture. [12] Some experts further recommend osteoporosis treatment in premenopausal women who need a long-term glucocorticoids therapy with a significant decline in BMD for age or a Z-score below -2.0.[15] Given the low proportions of fracture diagnosis, glucocorticoids use, and BMD examination among bisphosphonates initiators in our study, there may be a gap between the current guidelines and clinical practice.

Strengths of this study include the large population-based cohort of bisphosphonates initiators and the relatively long and recent study period to observe the temporal trend in bisphosphonates use. This study also has several limitations. First, the study was not designed to determine the reasons for a decrease in bisphosphonate use. Second, even though we used a year prior to bisphosphonate initiation to assess clinical characteristics including potential indications for bisphosphonate treatment, the 1-year time period may not be sufficiently long. Third, there is potential under ascertainment of other indications for bisphosphonates, such as giant cell lesions of the jaw, fibrous dysplasia, Gaucher’s disease and other uncommon metabolic bone disease due to incomplete recording of these diagnoses in the claims database. Fourth, this study may not be generalizable because this study included a commercially insured population only. Lastly, we are unable to check whether patients underwent a BMD testing if they did not claim the insurer for the test.

In conclusion, this study shows that use of bisphosphonates in patients aged 10–45 was uncommon and significantly decreased over the past decade in the U.S. This study also suggests a potential gap in understanding clinical practice patterns in treating young patients at a possible risk for osteoporosis, as some bisphosphonate initiators were with no clear indication for bisphosphonate treatment recorded in the year prior to initiating a bisphosphonate. The reason for the decline in the use of bisphosphonates should be further examined, as it cannot be explained by the change in the prevalence of potential risk factors for osteoporosis in the population. Furthermore, there is a need to evaluate factors determining bisphosphonates initiation among young patients including women of childbearing age and to assess the effectiveness and safety of bisphosphonates in the under-studied population.
